# Clinical-Pathological and Molecular Evaluation of 451 NIFTP Patients from a Single Referral Center

**DOI:** 10.3390/cancers14020420

**Published:** 2022-01-14

**Authors:** Paola Vignali, Agnese Proietti, Elisabetta Macerola, Anello Marcello Poma, Liborio Torregrossa, Clara Ugolini, Alessio Basolo, Antonio Matrone, Teresa Rago, Ferruccio Santini, Rossella Elisei, Gabriele Materazzi, Fulvio Basolo

**Affiliations:** 1 Department of Surgical, Medical, Molecular Pathology and Critical Area, University of Pisa, 56126 Pisa, Italy; paola.vignali@phd.unipi.it (P.V.); a.proietti@ao-pisa.toscana.it (A.P.); elisabetta.macerola@for.unipi.it (E.M.); marcello.poma@med.unipi.it (A.M.P.); l.torregrossa@ao-pisa.toscana.it (L.T.); clara.ugolini@unipi.it (C.U.); gabriele.materazzi@med.unipi.it (G.M.); 2 Department of Clinical and Experimental Medicine, University of Pisa, 56126 Pisa, Italy; alessio.basolo@med.unipi.it (A.B.); antonio.matrone@med.unipi.it (A.M.); rago@endoc.med.unipi.it (T.R.); ferruccio.santini@med.unipi.it (F.S.); rossella.elisei@med.unipi.it (R.E.)

**Keywords:** thyroid tumours, NIFTP, molecular pathology, FNA, cytology

## Abstract

**Simple Summary:**

Non-invasive follicular thyroid neoplasms with papillary-like nuclear features (NIFTP) are follicular thyroid neoplasm with an indolent clinical behaviour. In this study, we evaluated a retrospective series of 451 NIFTPs, of which 197 (43.7%) presented in coexistence with collateral thyroid tumours. Unifocal and multifocal NIFTPs did not show peculiar ultrasound, cytological, molecular, and histo-pathological characteristics. Considering the high rate of coexisting carcinomas, NIFTP patients might benefit from monitoring of the contralateral lobe.

**Abstract:**

Background: Non-invasive follicular thyroid neoplasms with papillary-like nuclear features (NIFTPs) were introduced in thyroid pathology in 2016. NIFTPs are a group of follicular neoplasm with an indolent behaviour. In this study, we gathered a large retrospective cohort of NIFTPs and compared those presenting as solitary lesions and NIFTPs found in multifocal setting. Methods: A retrospective search of NIFTPs was performed, and the clinico-pathological features were recorded. For a subgroup of patients, pre-surgical ultrasound (US) evaluation, cytological diagnosis, and molecular analysis were available. Results: We collected 451 NIFTPs; 254 (56.3%) were truly solitary tumours, while 197 coexisted with one or more NIFTP/cancer. Contrasting unifocal and multifocal settings, NIFTPs size was the only significantly different parameter. Preoperatively, NIFTP nodules mostly showed low-risk US characteristics, indeterminate cytology and a RAS-like molecular profile. Conclusion: NIFTPs often coexist with collateral thyroid tumours. However, no clinical-pathological differences can be observed between solitary and “multifocal” NIFTPs. Despite the well-established clinical indolence of NIFTP, a careful monitoring of the contralateral lobe should not be excluded.

## 1. Introduction

Since 2016, a new histological entity was introduced in the classification of thyroid tumours: non-invasive follicular thyroid neoplasms with papillary-like nuclear features (NIFTPs) made their first appearance in thyroid pathology [1]. NIFTPs were previously classified as encapsulated follicular variant papillary thyroid carcinoma (FVPTC). FVPTCs represent 30% of papillary thyroid carcinoma (PTC) [2]; they show the nuclear characteristics typical of PTC, but an almost exclusive follicular growth pattern [3]. FVPTCs can be divided in two main subtypes: infiltrative and encapsulated (with or without invasion of tumour capsule) [4]. Infiltrative FVPTCs can show recurrence, have a metastatic potential, and a BRAF mutation frequency of about 25% [5]. In contrast, encapsulated FVPTCs show lower risk of recurrence and lymph node metastases, with a high prevalence of *RAS* mutations and lack of *BRAF^V600E^* mutations [6,7]. It has been demonstrated that these tumours, when no capsular nor vascular invasion is present, have an excellent prognosis after complete resection, even when treated only with lobectomy [8,9]. Therefore, based on their low-risk molecular profile and indolent biological behaviour, they should be distinguished from infiltrative FVPTCs [5,10]. To reduce clinical overtreatment and psychological consequences associated with a diagnosis of “cancer”, non-invasive encapsulated FVPTCs have been reclassified as NIFTPs [1], with the goal to denote a group of follicular neoplasm with an indolent behaviour [11]. The diagnosis of NIFTP is only possible after surgical excision of the nodule, because the tumour capsule must be entirely inspected to exclude invasion foci. Moreover, a preoperative NIFTP diagnosis is extremely difficult, as they do not show peculiar ultrasound (US) nor cyto-morphological features [12]. On fine-needle aspiration (FNA), they are mostly classified as indeterminate nodules [13].

From the molecular point of view, as reported by TCGA, PTCs can be divided into *BRAF^V600E^*-like and *RAS*-like [14] tumours, and NIFTPs are unequivocally *RAS*-like lesions [15]. NIFTPs harbour *RAS* mutations in 40–70% of cases, while *BRAF^V600E^* mutation should not be detected in NIFTPs [16]. *BRAF*^K601E^ can be present, and has been detected in 2–11.5% of cases. Furthermore, NIFTPs harbour gene fusions in *PPARG* and *THADA* genes, typically detected in *RAS*-like tumours, with a prevalence of 22% and 40%, respectively [16]. Therefore, the molecular profile of NIFTP does not allow a definitive preoperative identification.

In the present study, a large mono-institutional series of NIFTP has been retrospectively evaluated. The aim was to investigate clinico-pathological characteristics of NIFTP with a particular focus on the multifocal scenario, which represent a controversial issue. Potential differences between NIFTPs as solitary tumours and in multifocal setting have been also investigated, taking into account US, cytology, and molecular NIFTP features.

## 2. Materials and Methods

A retrospective search of non-invasive follicular thyroid neoplasm with papillary-like nuclear features (NIFTP) was performed in the database of the Surgical Pathology Section of the University Hospital of Pisa from January 2016 to December 2020. A search of the term “NIFTP” was used for the selection of cases. The entire series of haematoxylin-eosin slides of all cases were reviewed by three pathologists (F.B., A.P., and L.T.) with experience in thyroid pathology, according to the current World Health Organization (WHO) criteria [4]. The following clinico-pathological features of NIFTP were recorded: tumour size, nuclear score, percentage of solid areas, and presence of multifocal disease, i.e., NIFTP coexisting with one or more NIFTP/carcinoma. In this last case, the size and histotype of collateral carcinoma were specified; moreover, the clinically most relevant nodule (index nodule) was identified based on clinical information reported on the pathological report.

For a subgroup of patients, pre-surgical ultrasound (US) evaluation, cytological diagnosis, and molecular analysis were available.

Neck US examination was performed by Real-time instrument (Technos, Esaote Biomedica, Genova, Italy) with a 7.5-MHz linear transducer [17]. Characteristics of nodules in terms of nodule composition, borders, echogenicity, shape, and presence of microcalcifications were recorded. Nodules were scored according to the EU-TIRADS system [18].

Cytological diagnosis was performed following both the Bethesda System for Reporting Thyroid Cytopathology (TBSRTC) [19] and the Italian Consensus for Classification and Reporting of Thyroid Cytology (ICCRTC) [20].

Molecular analysis was performed on formalin-fixed, paraffin-embedded tissue of patients whose cytological evaluation was performed in our institution, and biological material was available DNA was purified by using the Qiamp DNA Mini kit (Qiagen, Hilden, Germany). One paraffin block was selected for each case, and 2 to 6 10 µm-tick sections were obtained. After standard deparaffinization and rehydration of sections, tumour areas were collected by manual macrodissection and placed in a tube containing the lysis buffer and proteinase-K. All subsequent procedures for nucleic acids purification were performed following the protocol provided by the manufacturer. DNA was analysed for *RAS* family mutations exon 3 (*NRAS*, *HRAS*, and *KRAS*) and *BRAF* exon 15 by using high-resolution melt analysis (Type-it HRM PCR Kit, Qiagen) followed by direct sequencing.

Continuous variables are presented as median and interquartile range (IQR) and were analysed by the Mann–Whitney U test. The association between categorical variables were assessed by the Chi-square test or the Fisher exact test whenever appropriate. The analyses were carried out in R environment (https://www.r-project.org/, version 4.1.2, last accessed on 25 November 2021).

## 3. Results

**Figure 1:**
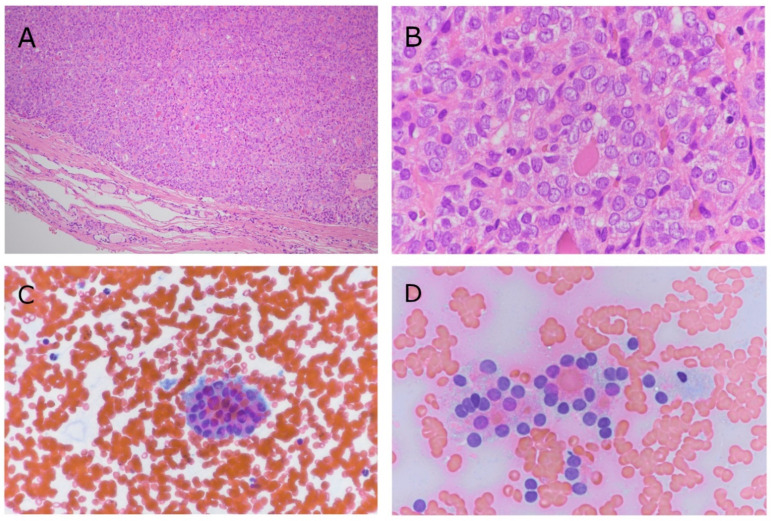
Histopathological and cytological images of NIFTPs. (**A**) Low magnification (original magnification: 10×). The tumour appears completely encapsulated without signs of vascular and/or capsular invasion. (**B**) Higher magnification (60×) of neoplastic cells. Papillary-like nuclear features are evident at this magnification. (**C**) Cytological smear of the same case diagnosed as indeterminate nodule (60×). The picture shows a microfollicular structure with mild/moderate nuclear atypia. (**D**) Another NIFTP case with indeterminate cytology. Two microfollicles are reported at high magnification (60×).

A total of 451 patients with NIFTP were retrieved. There were 321 (71.2%) female and 130 (28.8%) male patients. The median age was 50 years (IQR 40–59). The clinico-pathological characteristics of NIFTPs are summarized in Table 1. The median size of the lesion was 1.7 cm (IQR 0.9–3.0); 148 (32.8%) patients had microNIFTP (≤1 cm). The majority of lesions (85.4%) had a nuclear score of 2 and 14.6% of 3. There were no solid areas in 286 (63.4%) lesions, while in 165 (36.6%) lesions, solid areas were present with a percentage lower than 30% (Figure 1). In 186 cases (41.2%), NIFTP was in the context of normal parenchyma (solitary nodule). In the remaining cases, it was found in the context of multinodular thyroid parenchyma (58.8%). NIFTP size was significantly larger in solitary cases (*p*-value < 0.0001).

A total of 197 NIFTPs (43.7%) presented with one or more collateral NIFTP/malignant thyroid tumours, as shown in Table 2. NIFTP that coexisted with collateral malignant tumours were significantly smaller (*p*-value < 0.001), and total thyroidectomy rate was significantly higher (*p*-value < 0.0001). Among patients with collateral malignant lesions submitted to hemithyroidectomy, four needed repeated surgery for completion thyroidectomy. In the contralateral lobe, histological examination showed a microPTC in one patient.

**Figure 2:**
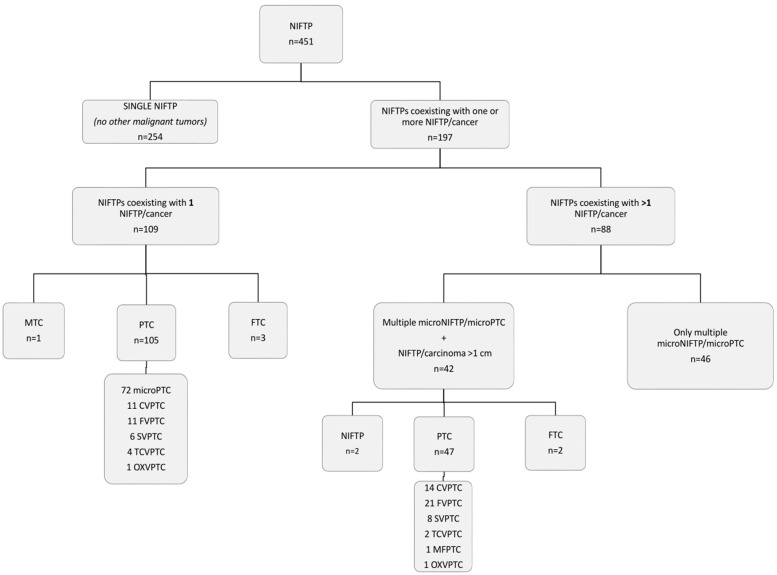
Detailed distribution of the 451 NIFTPs with or without other coexisting thyroid lesions. Abbreviations: NIFTP, non-invasive follicular thyroid neoplasm with papillary-like features, MTC, medullary thyroid carcinoma, PTC, papillary thyroid carcinoma, FTC, follicular thyroid carcinoma, CVPTC, classical-variant of papillary thyroid carcinoma, FVPTC, follicular-variant of papillary thyroid carcinoma, SVPTC, solid-variant of papillary thyroid carcinoma, TCVPTC, tall-cell variant papillary thyroid carcinoma, and OXVPTC, oxyphilic-variant of papillary thyroid carcinoma.

The histotype of malignant tumours coexisting with NIFTP is reported in Table 3. In multifocal disease setting, there were predominantly papillary thyroid cancers (PTC), mainly microPTCs. Detailed distribution of coexisting NIFTPs/cancers is shown in Figure 2.

NIFTP was the index lesion in 117 out of 197 multiple cases (59.4%); in 61 cases (31.0%) another malignant lesion was the index nodule; in 19 cases (9.6%) the index nodule was a benign thyroid nodule. When NIFTP was not the index lesion, its size was ≤1 cm in 50 out of 80 cases (62.5%). When NIFTP was the index lesion, the collateral malignant tumour was ≤1 cm in 102 out of 117 cases (87.2%). In 15 cases (12.8%), the collateral malignant carcinoma was greater than 1 cm in diameter. NIFTP considered as index nodules were significantly larger (*p*-value < 0.0001).

For a subset of NIFTP cases, information on US features, presurgical cytological diagnosis, and molecular results was available (Table 4). US characteristics were low-risk in the majority of nodules (solid composition in 95.3%; well-defined borders in 96.5%; absence of microcalcifications in 97.7%; isoechoic pattern in 62.8%; wider-than-tall in 94.2%; and EU-TIRADS 3 category in 96.5% of cases). As regards multifocality, 40 out of 86 nodules (46.5%) on histology were single NIFTPs, while 46 out of 86 nodules (53.5%) were NIFTPs coexisting with collateral malignant tumours. US features were not significantly different according to multifocality.

The most represented cytological category was the Bethesda III (TIR3A), in 91 out of 184 nodules (49.5%).

Material to perform molecular analysis was available in 137 cases. As shown in Table 4, 62 NIFTP out of 137 (45.3%) were mutated. The majority of NIFTP harboured *RAS* mutations (40.1%), while only 5.1% of NIFTP harboured non-V600E *BRAF* mutations: four were mutated in codon 601 (p.K601E), one had a deletion (p.T599del), one had an insertion (p.A598_T599insV) and one had a delins (p.V600_S605delVKSRWSinsD). NIFTP mutational status showed no statistical associations with cytological categories nor US features; moreover, there were no associations between the presence of mutations and NIFTP histological features, including solitary/multifocal tumours.

## 4. Discussion

Non-invasive follicular thyroid neoplasms with papillary-like features (NIFTP) have been in the classification of thyroid cancers since 2016 [1]. NIFTP is a follicular cell-derived non-invasive neoplasm with a follicular growth pattern and nuclear features of papillary thyroid carcinoma (PTC) that has an extremely low malignant potential [4]. According to the latest guidelines of the World Health Organization, NIFTP is a solitary, well-demarcated nodule, typically with a thin to moderately thick capsule. The tumours are usually 2–4 cm in size but can be much larger.

Over the last 5 years, several clinico-pathological aspects related to NIFTP have been investigated.

Xu and colleagues tried to investigate whether also subcentimeter, non-invasive encapsulated follicular variant (NI-EFV) PTC could be renamed as NIFTP. They collected 52 patients with unifocal microPTC NI-EFV, with a control group of invasive microPTC (*n* = 52). They did not observe recurrence in the group of microPTC NI-EFV, while in the control group there were cases of nodal metastasis and recurrence. Subcentimeter NI-EFV PTC were characterized by extremely indolent clinical course, and should be considered as NIFTP [21]. The same group evaluated a series of non-invasive encapsulated FVPTC with oncocytic features, and confirmed their indolent clinical behaviour [22]. Recently, the importance of the complete tumour encapsulation has been also highlighted in classical PTCs [23].

The concept of solitary NIFTP, intended as a single thyroid nodule in the context of normal parenchyma, has been also questioned. Some authors tried to address the issue of multifocal and collateral disease in the context of NIFTP. Canberk and colleagues collected a series 74 NIFTP (including lesions <1 cm in size), of which 61 were solitary (82.4%) and 13 were multifocal (17.6%), specifically, 11 were PTC and 2 were NIFTPs. They demonstrated that multifocal lesions tend to be associated with an index lesion with smaller size, and their findings reinforce previous suggestions that multifocal disease is part of the spectrum of NIFTP neoplasm [24,25]. Canini and colleagues collected 68 NIFTP in a 9 year-long period, including nodules smaller than 1 cm. In their study, 41 NIFTP (60.1%) were in a context of multinodular parenchyma: a significant proportion of these NIFTPs were incidental (29%). In 10 out of 68 cases (14.7%), NIFTPs were in coexistence with a thyroid carcinoma. These authors suggest that close monitoring of the contralateral lobe in patients with NIFTP not submitted to total thyroidectomy should be performed [26]. Song and colleagues collected 87 NIFTP cases from 82 patients. In 38 patients (46.3%), 75 malignant tumours were identified, mostly microPTCs [27]. Seo and colleagues collected 238 cases of NIFTPs, of which 64 (26.7%) coexisted with other malignant thyroid tumours [28]. On the other hand, Turan and Ozkara retrospectively evaluated 481 FVPTCs, of which 84 (3.9%) matched a diagnosis of NIFTP. Most of them were solitary NIFTPs, while 15 were multifocal NIFTPs (17.9%); cases in which NIFTP coexisted with other thyroid malignancies were excluded [29].

Herein, we have collected the largest retrospective and monocentric series of patients with diagnosis of NIFTP (*n* = 451), which were included independently of the presence of coexisting thyroid tumours. In our study, 197 out of 451 NIFTP (43.7%) were in coexistence with one or more collateral NIFTP or malignant lesions. The size of multiple NIFTPs size was significantly smaller compared to that of solitary ones. Total thyroidectomy rate was significantly higher in NIFTPs that coexisted with one or more collateral thyroid lesions, likely because they presented more frequently with multinodular parenchyma.

Interestingly, among 197 cases of NIFTP coexisting with other malignant lesions, collateral lesions were present both in the same lobe (55.3%) and in the contralateral lobe (44.7%). As shown in Figure 2, in 109 cases, NIFTP coexisted with only one other NIFTP or carcinoma; in 88 cases, NIFTP coexisted with multiple NIFTPs or carcinomas. The coexistence of multiple NIFTPs, as well as NIFTP with one collateral thyroid malignancy, has been already described with a frequency ranging from 14.7 to 46.3% [24,25,26,27,28]. However, the fact that in 88 cases of our series one NIFTP presented with multiple thyroid carcinomas in the same patient represents an unexpected finding, which further complicates the controversy of how a NIFTP should be named and managed in the multifocal setting. Another interesting observation was that, in 46 out of 88 patients, NIFTP coexisted with only multiple microNIFTP and/or microPTC. MicroPTCs are quite common in the general population, and often represent an incidental finding [30]. The American Thyroid Association (ATA) included unifocal or multifocal microPTC as low-risk tumours, unless extrathyroidal extension is present [31]. However, in recent years, the optimal management of microPTC has been subjected to discussion, and further risk-stratification of microPTC seems necessary [30,32].

Moreover, in a considerable number of patients (42 out of 88, 9.3% of the entire cohort), NIFTP was in the presence of carcinomas greater than 1 cm, the majority being PTCs. These tumours, clearly guided clinical patients’ management independently of the presence of NIFTP, which have different therapeutic indications and a likely-benign behaviour. In this context, it should be highlighted that in 117 out of 197 “multifocal” cases, NIFTP was considered the index nodule (59.4%); in 102 cases, the collateral lesion was a microNIFTP/microPTC, while, in the remaining 15 cases (12.8%), the collateral carcinoma was greater than 1 cm. Therefore, in this group of cases, the nodule corresponding to the NIFTP would have guided the management of patients.

Furthermore, in our series, NIFTP had a very often a preoperative indeterminate cytology (Bethesda III, TIR3A). These data are in line with previous studies, that underline the morphological overlap between NIFTP and other follicular neoplasms including papillary thyroid carcinoma [33]. Therefore, even performing a careful examination, NIFTPs cannot be further characterized on cytology. Similarly, the molecular profile of NIFTPs overlaps to that of follicular thyroid lesions, i.e., mostly *RAS* mutations.

Our study presents some limitations. One of these is the retrospective nature of the study. Moreover, our findings including US, cytological, and molecular data were limited to a subgroup of the NIFTP cohort. Finally, the mutational screening of nodules included only part of the spectrum of *RAS*-like mutations, while alterations in genes such as *THADA* and *PPARG* were not tested.

## 5. Conclusions

To sum up, NIFTP can be found as a solitary thyroid nodule or in coexistence with other thyroid nodules, either benign or malignant. In our series, no significant differences were found between single and multiple NIFTPs in terms of clinico-pathological characteristics, with the exception of NIFTP size.

Considering the high rate of coexisting carcinomas, and despite the clinical indolence of NIFTP, a careful monitoring of the contralateral lobe should not be excluded.

## Figures and Tables

**Table 1:** Description of 451 NIFTP cases.

NIFTP Clinico-Pathological Characteristics	N (%)
Gender	
Female	321 (71.2)
Male	130 (28.8)
Age, median (IQR)	50 (40–59)
Size [cm], median (IQR)	1.7 (0.9–3.0)
microNIFTP (≤1 cm)	148 (32.8)
Nuclear Score	
2	385 (85.4)
3	66 (14.6)
Solid areas	
Absent	286 (63.4)
Present (<30%)	165 (36.6)
Collateral thyroid parenchyma	
Normal	186 (41.2)
Multinodular	265 (58.8)

Abbreviations: NIFTP, non-invasive follicular thyroid neoplasm with papillary-like nuclear features; IQR, interquartile range.

**Table 2:** Characteristics of NIFTP with and without collateral malignant lesions.

Variation	NIFTP N = 451		
	Absence of Collateral Malignant Lesions N = 254 (56.3%)	Presence of Collateral Malignant Lesions N = 197 (43.7%)	*p*-Value
Gender			
Female	179 (70.5)	142 (72.1)	0.79
Male	75 (29.5)	55 (27.9)	
Age, median (IQR)	50 (39–59)	51 (43–59)	0.44
Size [cm], median (IQR)	2 (1.0–3.3)	1.4 (0.8–2.5)	<0.0001
Nuclear score			0.86
2	218 (85.8)	167 (84.8)	
3	36 (14.2)	30 (15.2)	
Solid areas			0.61
Absent	158 (62.2)	128 (65.0)	
Present (<30%)	96 (37.8)	69 (35.0)	
Localization of collateral malignant lesions			
Ipsilateral	/	109 (55.3)	
Contralateral	/	88 (44.7)	
Type of surgery			
Total thyroidectomy	181 (71.3)	174 (88.3)	<0.0001
Lobectomy	73 (28.7)	23 (11.7)	

Abbreviations: NIFTP, non-invasive follicular thyroid neoplasm with papillary-like features; IQR, interquartile range.

**Table 3:** Histotype of the malignant lesions found in coexistence with NIFTPs in 197 patients. MicroPTC have not been included in this table.

Histological Type	*N* (%)
PTC	80 (93.0)
FVPTC	32 (40.0)
CVPTC	25 (31.3)
SVPTC	14 (17.5)
TCVPTC	6 (7.5)
OXPTC	2 (2.5)
MFPTC	1 (1.2)
FTC	5 (5.8)
MTC	1 (1.2)

Abbreviations: PTC, papillary thyroid carcinoma, FVPTC, follicular-variant of papillary thyroid carcinoma, CVPTC, classical-variant of papillary thyroid carcinoma, SVPTC, solid-variant of papillary thyroid carcinoma, TCVPTC, tall-cell variant papillary thyroid carcinoma, OXVPTC, oxyphilic-variant of papillary thyroid carcinoma, MFPTC, macro follicular papillary thyroid carcinoma, FTC, follicular thyroid carcinoma, and MTC, medullary thyroid carcinoma.

**Table 4:** Subset of NIFTP cases with available cytological diagnosis, ultrasound data, and molecular analysis.

Presurgical US Characteristics of NIFTPs *n* = 86			N (%)
Nodule composition			
Solid			82 (95.3)
Mixed			3 (3.5)
Spongiform			1 (1.2)
Borders			
Well-defined			83 (96.5)
Lobulated or irregular			3 (3.5)
Microcalcifications			
Yes			2 (2.3)
No			84 (97.7)
Echogenicity			
Isoechoic			54 (62.8)
Hyperechoic			3 (3.5)
Hypoechoic			28 (32.5)
Anechoic			1 (1.2)
Shape			
Wider-than-tall			81 (94.2)
Taller-than-wide			3 (3.5)
Missing data			2 (2.3)
EU-TIRADS			
2			3 (3.5)
3			83 (96.5)
Cytology *n* = 184	N (%)		N (%)
TBSRTC		ICCRTC	
I	10 (5.4)	TIR1	10 (5.4)
II	34 (18.5)	TIR2	34 (18.5)
III	91 (49.5)	TIR3A	92 (50.0)
IV	42 (22.8)	TIR3B	41 (22.3)
V	6 (3.3)	TIR4	6 (3.3)
VI	1 (0.5)	TIR5	1 (0.5)
Molecular Analysis *n* = 137		N Mutated (%)	
Ras family genes mutations			
NRAS		35 (25.5)	
HRAS		12 (8.8)	
KRAS		8 (5.8)	
non-V600E BRAF mutations		7 (5.1)	
Total		62 (45.3)	

Abbreviations: TBSRTC, the Bethesda system for reporting thyroid cytopathology, ICCRTC, Italian consensus for classification and reporting of thyroid cytology, US, ultrasound; and NIFTP, non-invasive follicular thyroid neoplasm with papillary-like features.

## Data Availability

All data generated or analysed during this study are included in this manuscript.
